# Prevalence and Correlation of Gastrointestinal Symptoms With Glycemic Control in Pediatric Patients With Type 1 Diabetes Mellitus: A Cross-Sectional Study

**DOI:** 10.7759/cureus.93397

**Published:** 2025-09-28

**Authors:** Bakr A Jalawi, Mohammed H Alatawi, Khaled Alqoaer, Waad Alotaibi

**Affiliations:** 1 Medicine, King Salman Armed Forces Hospital, Tabuk, SAU; 2 Pediatrics, King Salman Armed Forces Hospital, Tabuk, SAU; 3 Pediatric Gastroenterology, King Salman Armed Forces Hospital, Tabuk, SAU

**Keywords:** diet compliance, gastrointestinal symptoms, glycemic control, hba1c, pediatric diabetes, type 1 diabetes mellitus

## Abstract

Background: Type 1 diabetes mellitus (T1DM) is the most common form of diabetes in children and adolescents. Gastrointestinal (GI) symptoms, including abdominal pain, diarrhea, nausea, and vomiting, are increasingly recognized as complications that may worsen disease management.

Objective: This study aims to determine the prevalence of GI symptoms in pediatric patients with T1DM and to assess their correlation with glycemic control.

Materials and methods: A cross-sectional observational study was conducted over a six-month period at King Salman Armed Forces Hospital, Tabuk, Saudi Arabia. Fifty-four patients aged 3-14 years with T1DM were evaluated. Data on GI symptoms and glycemic control, categorized by HbA1c (<7% vs ≥7%), were collected from electronic medical records and analyzed using descriptive statistics, Chi-square tests, and Fisher’s exact tests.

Results: GI symptoms were reported in 88.9% of patients, with a prevalence of 97.9% among those with uncontrolled diabetes, compared to 28.6% in those with controlled diabetes. Diarrhea (77.8%) and abdominal pain (66.7%) were the most frequent symptoms. Elevated HbA1c was significantly associated with abdominal pain (p = 0.002) and diarrhea (p = 0.001). Diet compliance correlated strongly with glycemic control (p = 0.001).

Conclusions: GI symptoms are highly prevalent in children with poorly controlled T1DM. Regular screening for GI complaints and early intervention are essential to improve glycemic outcomes and quality of life. Longitudinal studies are required to clarify the causal relationship between glycemic control and GI symptoms.

## Introduction

Type 1 diabetes mellitus (T1DM) is the most common form of diabetes in children and adolescents, accounting for over 90% of all diabetes cases in this population [1]. It is an autoimmune disorder characterized by the destruction of insulin-producing beta cells in the pancreas, resulting in lifelong dependence on exogenous insulin therapy. The global incidence of T1DM in children has been increasing in recent years, with the highest rates observed in Northern Europe, North America, and Australia. This rise is concerning due to the significant long-term gastrointestinal (GI) complications [2].

GI symptoms in pediatric patients with T1DM are increasingly recognized as a significant issue, affecting quality of life and complicating disease management. These symptoms, which range from mild discomfort to severe conditions requiring specialized care, commonly include abdominal pain, nausea, vomiting, and altered bowel habits, further complicating glycemic control [3]. Children often report upper and lower abdominal pain, while adults more frequently experience lower GI symptoms. Notably, patients with GI symptoms tend to have higher HbA1c levels and a greater likelihood of microvascular complications. However, no association has been found between GI symptoms and autoimmune conditions like thyroid disease or celiac disease (CD) [4].

One of the most significant GI complications in pediatric patients with T1DM is gastroparesis, a condition characterized by delayed gastric emptying [5]. Pediatric gastroparesis is particularly challenging because it can lead to unpredictable blood glucose levels, making insulin management difficult. Studies have shown that gastroparesis can occur even in children with relatively short disease durations, highlighting the importance of early detection and management [6].

Another critical GI condition associated with pediatric patients with T1DM is CD, an autoimmune disorder triggered by gluten ingestion. CD is significantly more common in pediatric patients with T1DM compared to the general pediatric population. Estimates indicate that up to 20% of pediatric patients with T1DM may also have CD. The prevalence of CD in this population ranges from 5.5% to 20% based on serological testing and from 1.6% to 16.4% based on histopathological findings, underscoring the importance of screening and early diagnosis in managing these comorbid conditions effectively [7]. This association poses significant challenges in dietary management, as these children require both strict insulin regimens and gluten-free diets, which can be challenging to adhere to, especially during childhood and adolescence.

Exocrine pancreatic insufficiency, characterized by an insufficient production of digestive enzymes, has also been observed in children with T1DM. This condition leads to malabsorption of nutrients, resulting in growth and developmental delays if not adequately managed [8]. The recognition of exocrine pancreatic insufficiency in pediatric patients with T1DM is essential for early intervention and treatment, as it directly impacts nutritional status and overall health outcomes.

Furthermore, recent research has investigated the role of the gut microbiota in children with T1DM. Results suggest that alterations in the gut microbial composition may influence both the development of T1DM and the onset of GI symptoms. This emerging area of research holds promise for developing novel therapeutic strategies aimed at restoring gut microbiota balance and mitigating GI symptoms in pediatric patients with T1DM [9,10].

Given the limited research in this area, our study aims to investigate the prevalence of GI symptoms in pediatric patients with T1DM and explore any potential correlation between these symptoms and glycemic control. This retrospective observational study aims to provide crucial insights that can inform more effective management practices for children with T1DM.

## Materials and methods

Methodology

This cross-sectional observational study aimed to evaluate the prevalence of GI symptoms and their association with glycemic control in pediatric patients diagnosed with T1DM.

Study design and population

Our research was conducted over a six-month period at King Salman Armed Forces Hospital, Tabuk, Saudi Arabia. The Academic Affairs Research Ethics Team issued approval KSAFH-RET-2024-565. The study population comprised children aged between three and 14 years who were diagnosed with T1DM and had no prior history of GI diseases. The inclusion criteria focused on children within the specified age range who had been diagnosed with T1DM between 2018 and 2023. Exclusion criteria included children younger than three years, those older than 14 years, and females who had begun menstruation.

Sampling methods and data collection

The sampling method employed in this study was a non-probability convenience sampling technique. Eligible patients who met the inclusion criteria and were admitted through the emergency room or seen in outpatient clinics during the study period were included in the analysis. Data were collected retrospectively from the patients’ electronic medical records, encompassing demographic details, clinical presentations, laboratory results, and the presence of GI symptoms. Glycemic control was assessed using the most recent HbA1c levels, categorizing patients into two groups: controlled (HbA1c <7%) and uncontrolled (HbA1c ≥7%).

Statistical analysis

Statistical analyses were performed using SPSS Statistics version 25 (IBM Corp. Released 2017. IBM SPSS Statistics for Windows, Version 25.0. Armonk, NY: IBM Corp.). Descriptive statistics were used to summarize patient demographics and clinical characteristics. The prevalence of GI symptoms was calculated, and the association between GI symptoms and glycemic control was assessed using chi-square tests and Fisher’s exact tests. Chi-square tests were used to assess the association between categorical variables, while Fisher’s exact tests were applied where sample sizes were small. A p-value of less than 0.05 was considered statistically significant.

## Results

The study included a total of 54 pediatric patients diagnosed with T1DM, with a mean age of 9.2 ± 3.2 years. The age distribution within the sample was as follows: 27.8% (n = 15) of patients were between three and six years old, 31.5% (n = 17) were aged seven to 10 years, and 40.7% (n = 22) were between 11 and 14 years old. The gender distribution was skewed toward females, who constituted 61.1% of the sample (n = 33), while males made up 38.9% (n = 21). A significant portion of patients, 57.4% (n = 31), were noncompliant with their dietary recommendations, and 81.5% (n = 44) experienced weight loss (Table 1).

**Table 1:** Demographic and clinical characteristics of pediatric patients with T1DM T1DM: type 1 diabetes mellitus

Variable	Value	Percent
Age 3-6 years	15	27.8
Age 7-10 years	17	31.5
Age 11-14 years	22	40.7
Male	21	38.9
Female	33	61.1
Diet compliance - yes	23	42.6
Diet compliance - no	31	57.4
Weight gain	10	18.5
Weight loss	44	81.5
HbA1c normal	7	13
HbA1c abnormal	47	87
Vitamin D normal	29	93.5
Vitamin D abnormal	2	6.5
Hemoglobin low	3	5.6
Hemoglobin normal	37	68.5
Hemoglobin high	14	25.9
Albumin low	1	1.9
Albumin normal	53	98.1
Lantus	1	1.9
Metformin	1	1.9
Toujeo	2	3.7
Tresiba	50	92.6

**Figure 1:**
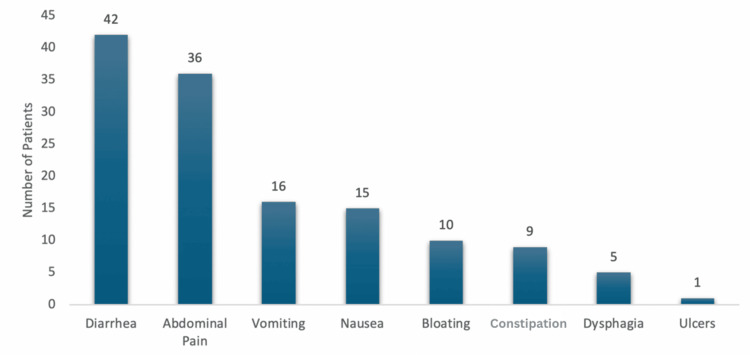
Prevalence of GI symptoms in pediatric patients with T1DM Statistical analysis was performed using chi-square and Fisher’s exact tests; p < 0.05 was considered significant. GI: gastrointestinal, T1DM: type 1 diabetes mellitus

The overall prevalence of GI symptoms within the study cohort was high (88.9%), indicating that nearly 89% of the patients experienced at least one GI symptom, with a marked difference observed between patients with controlled and uncontrolled diabetes. Specifically, 28.6% of patients with controlled diabetes (HbA1c <7%) exhibited GI symptoms, compared to a staggering 97.9% of those with uncontrolled diabetes (HbA1c ≥7%). This stark contrast underscores the significantly higher burden of GI symptoms among patients with poor glycemic control (Table 2, Figure 1).

**Table 2:** Association between glycemic control and GI symptoms in pediatric patients with T1DM Statistical analysis was performed using chi-square and Fisher’s exact tests; p < 0.05 was considered significant. GI: gastrointestinal, T1DM: type 1 diabetes mellitus

Symptom	Normal (n = 7)	Prevalence	Abnormal (n = 47)	Prevalence	p-value
Abdominal pain - yes	1	14.3%	35	74.5%	0.002
Abdominal pain - no	6		12		
Bloating - yes	0	0	10	21.3%	0.176
Bloating - no	7		37		
Dysphagia - yes	0	0	5	10.6%	0.365
Dysphagia - no	7		42		
Nausea - yes	0	0	15	32%	0.079
Nausea - no	7		32		
Diarrhea - yes	1	14.3%	41	87%	0.001
Diarrhea - no	6		6		
Vomiting - yes	0	0	16	34%	0.066
Vomiting - no	7		31		
Constipation - yes	0	0	9	19%	0.205
Constipation - no	7		38		
Ulcers - yes	0	0	1	2%	0.697
Ulcers - no	7		46		

The most frequently reported GI symptom was diarrhea, present in 77.8% (n = 42) of the patients, followed by abdominal pain, which was reported by 66.7% (n = 36) of the patients. Vomiting and nausea were also common, observed in 29.6% (n = 16) and 27.8% (n = 15) of the patients, respectively. Other symptoms, such as bloating, constipation, dysphagia, and ulcers, were less prevalent but still noteworthy, particularly in patients with uncontrolled diabetes (Figure 1).

Our research revealed significant associations between certain GI symptoms and glycemic control. A statistically significant relationship was found between abdominal pain and elevated HbA1c levels (p = 0.002). Among the 47 patients with uncontrolled diabetes, 74.5% (n = 35) reported experiencing abdominal pain, compared to only 14.3% (n = 1) of those with controlled diabetes. A similarly significant association was observed between diarrhea and uncontrolled diabetes (p = 0.001), with 87% (n = 41) of patients in the uncontrolled group reporting diarrhea, compared to only 14.3% (n = 1) of patients in the controlled group. Although other GI symptoms such as bloating, dysphagia, nausea, vomiting, and constipation were more frequently reported in patients with uncontrolled diabetes, these associations did not reach statistical significance (Table 2).

Diet compliance emerged as a critical factor associated with glycemic control, exhibiting a strong, statistically significant relationship (p = 0.001). All patients who adhered to dietary recommendations (n = 7) maintained normal HbA1c levels, whereas noncompliance was observed exclusively in patients with uncontrolled HbA1c levels (Table 3).

**Table 3:** Relationship between diet compliance, weight changes, and glycemic control Statistical analysis was performed using chi-square and Fisher’s exact tests; p < 0.05 was considered significant.

Variable	Normal	Abnormal	p-value
Diet compliance - yes	7	16	0.001
Diet compliance - no	0	31	
Weight gain	0	10	0.176
Weight loss	7	37	

After analyzing the relationship between diet compliance and HbA1c in different age groups, we found no significant association (p > 0.05). Specifically, among the three age groups (three to six years, 7 to 10 years, and 11 to 14 years), diet compliance and HbA1c levels did not differ significantly, with p-values of 0.233 and 0.341, respectively (Table 4).

**Table 4:** Relationship between age groups, diet compliance, and glycemic control Statistical analysis was performed using chi-square and Fisher’s exact tests; p < 0.05 was considered significant.

Variable	3-6 yrs	7-10 yrs	11-14 yrs	p-value
Diet compliance - yes	6	10	7	0.233
Diet compliance - no	9	7	15	
HbA1c normal	3	3	1	0.341
HbA1c abnormal	12	14	21	

Although weight loss was prevalent within the cohort, affecting 81.5% (n = 44) of patients, it did not demonstrate a significant association with glycemic control (p = 0.176) (Table 3). Laboratory assessments, including vitamin D, hemoglobin, and albumin levels, were also analyzed in relation to glycemic control. Although no statistically significant associations were found (p > 0.05), a trend was noted where patients with normal HbA1c levels generally had laboratory results within normal ranges (Table 5).

**Table 5:** Association between laboratory parameters and glycemic control Statistical analysis was performed using chi-square and Fisher’s exact tests; p < 0.05 was considered significant.

Variable	Normal	Abnormal	p-value
Vitamin D normal	4	25	0.574
Vitamin D abnormal	0	2	
Hemoglobin low	1	2	0.464
Hemoglobin normal	5	32	
Hemoglobin high	1	13	
Albumin low	0	1	0.697
Albumin normal	7	46	

## Discussion

The findings from our study highlight the significant prevalence of GI symptoms in pediatric patients with T1DM, particularly among those with poor glycemic control. Our study revealed that 88.9% of the patients experienced at least one GI symptom, with a marked difference observed between those with controlled and uncontrolled diabetes. Specifically, although only 28.6% of patients with controlled diabetes (HbA1c <7%) exhibited GI symptoms, an overwhelming 97.9% of those with uncontrolled diabetes (HbA1c ≥7%) did. These findings align with previous literature that associated poor glycemic control with an increased burden of GI symptoms in pediatric patients with T1DM.

Several studies have explored the relationship between T1DM and GI symptoms, underscoring the complex interplay between glycemic control and GI health. For example, a study by Lodefalk and Aman reported that GI symptoms are common in adolescents with T1DM; however, interestingly, the prevalence did not differ significantly from that of nondiabetic controls. However, in their study, GI symptoms were associated with factors such as female sex, poorer socioeconomic status, daily cigarette smoking, longer duration of diabetes, and irregular meal patterns, rather than with glycemic control alone​ [11]. This suggests that although glycemic control is a crucial factor, other variables may also contribute to the development and severity of GI symptoms in this population.

Our results showing a high prevalence of diarrhea (77.8%) and abdominal pain (66.7%) are consistent with findings from a study by Selbuz and Buluş, who observed that 54% of pediatric patients with T1DM reported at least one GI complaint, with gastroesophageal reflux and abdominal pain being the most common​ [12]. The strong association between these symptoms and poor glycemic control, particularly the significant correlation between elevated HbA1c levels and the presence of abdominal pain and diarrhea, underscores the importance of maintaining optimal glycemic control to mitigate GI complications.

However, our observation of the occurrence of GI symptoms with poor diabetic control raises a critical question: Does poor glycemic control lead to the development of GI symptoms, or do GI symptoms contribute to poor glycemic control? This study, being cross-sectional in nature, does not allow us to establish causality or determine the direction of this relationship. Factors such as the study’s retrospective design, the lack of longitudinal data, and potential confounding variables limit our ability to draw definitive conclusions. Understanding the direction of this relationship is significant because if GI symptoms indeed contribute to poor glycemic control, they could represent an important and modifiable target for intervention. Conversely, if poor glycemic control leads to the development of GI symptoms, this further emphasizes the need for stringent glycemic management in this population.

Interestingly, Vazeou et al. concluded that GI symptoms in children with type 1 diabetes are as frequent as in the general population and do not significantly impact diabetes control, suggesting that GI symptoms in patients with type 1 diabetes should be investigated and treated similarly to those in nondiabetic individuals [13]. This highlights the complexity of the relationship between GI symptoms and glycemic control and underscores the need for further research to determine whether the presence of these symptoms is merely coincidental or whether they actively contribute to glycemic instability. Future studies, particularly those employing longitudinal designs, are crucial for clarifying this relationship and informing effective clinical management.

Moreover, our study’s finding that the 11-14-year-old age group had a significant number of patients with poor diet compliance and uncontrolled diabetes, yet without statistical significance in the relationship between diet compliance, HbA1c levels, and age groups, is intriguing. The small sample size might explain this discrepancy within this age group, which could have reduced the power of the statistical tests. Additionally, unmeasured confounding factors, such as pubertal hormonal changes, psychological stress, or differences in diabetes management practices, could have influenced the outcomes. This observation aligns with findings by Ghazaiean et al., who reported that patients aged 10 to 14 years were at higher risk for suboptimal glycemic control, suggesting that this age group may require more targeted interventions​ [14].

In terms of specific GI disorders associated with T1DM, the literature indicates a significant overlap between T1DM and autoimmune conditions like CD and autoimmune gastritis (AIG). Our study did not specifically investigate the prevalence of CD or AIG; however, the high prevalence of GI symptoms in our cohort suggests that these conditions may be underlying factors. Kakleas et al. found that CD is more common in children with T1DM, with 8.6% of their study population testing positive for anti-tTG IgA, a marker for CD. The presence of CD in these patients was associated with younger age and shorter diabetes duration, highlighting the need for regular screening in this high-risk group​ [15]. Similarly, Meyers et al. described a case of AIG in a young adult with T1DM, emphasizing the importance of recognizing GI symptoms as potential indicators of comorbid autoimmune conditions​ [16].

The role of delayed gastric emptying, or gastroparesis, in pediatric patients with T1DM is another critical area of concern. Although our study did not directly measure gastric emptying times, the high prevalence of symptoms such as nausea and vomiting may be indicative of underlying motility disorders. Porter et al. conducted a pilot study using carbon-13 breath testing to measure gastric emptying in pediatric patients with T1DM and found that, although there was a trend toward delayed gastric emptying in these patients, the results were not statistically significant​ [17]. Some authors attributed the poor glycemic control to delayed gastric emptying, which causes a mismatch between the onset of insulin action and carbohydrate absorption [13,18]. However, it is important to note that gastric emptying delay does not occur in all patients with recurrent abdominal pain or chronic dyspepsia. This variability in gastric motility, even among those with significant GI symptoms, underscores the complexity of managing glycemic control in the presence of these disorders. This complexity aligns with our findings that similar symptoms were prevalent but not always associated with glycemic control, suggesting that further research with larger sample sizes is needed to explore this relationship more thoroughly.

Overall, our study contributes to the growing body of evidence that GI symptoms are highly prevalent in pediatric patients with T1DM, particularly those with poor glycemic control. The clinical implications are significant, as these symptoms can severely impact quality of life and complicate diabetes management. It is essential for clinicians to regularly assess GI symptoms in pediatric patients with T1DM and consider potential underlying conditions such as CD, AIG, or gastroparesis. Early detection and targeted interventions, including optimizing glycemic control and addressing specific GI disorders, are crucial in managing this vulnerable population effectively.

Future researchers should focus on longitudinal studies to better understand the causal relationships between glycemic control and GI symptoms in pediatric patients with T1DM. Additionally, investigating the role of gut microbiota, as suggested by emerging research, could open new avenues for therapeutic strategies aimed at reducing GI symptoms and improving overall outcomes in this population.

Limitations of the study

The primary limitations of this study include its cross-sectional design, which prevents the determination of causality between glycemic control and GI symptoms. Additionally, the sample size was relatively small, which may have limited the generalizability of the findings. The study relied on retrospective data from electronic medical records, which may have resulted in incomplete or inaccurate information. Additionally, the use of non-probability convenience sampling may introduce selection bias. As the study population consisted only of patients who presented to our hospital, the findings may not be fully generalizable to all children with T1DM in the broader community. Another limitation of this study is the unequal distribution of patients between the controlled and uncontrolled groups (n = 7 vs. n = 47). The small sample size in the controlled group limits the robustness of the statistical comparisons and increases the possibility that observed differences may be due to chance. Therefore, these findings should be interpreted cautiously. Incomplete laboratory data for some patients may have limited the power of certain analyses, and this should be considered when interpreting the results. Lastly, the study did not account for potential confounding variables such as dietary habits, socioeconomic status, and psychological factors, which could have influenced the observed outcomes.

## Conclusions

Our study highlights the high prevalence of GI symptoms in pediatric patients with T1DM, especially among those with poor glycemic control. The significant association between elevated HbA1c levels and symptoms such as diarrhea and abdominal pain underscores the critical impact of glycemic control on GI health. While our findings emphasize the need for regular assessment and targeted management of GI symptoms in these patients, they also raise important questions about whether poor glycemic control leads to GI symptoms or vice versa, questions that future longitudinal studies must address. Early detection and intervention remain crucial for improving both the quality of life and glycemic outcomes in pediatric patients with T1DM.
